# Metabolic dysfunction-associated steatotic liver disease predicts graft failure in kidney transplant recipients

**DOI:** 10.1093/ckj/sfag294

**Published:** 2026-08-31

**Authors:** Kyungho Lee, Yebin Park, Hojin Jeon, Junseok Jeon, Jung Eun Lee, Wooseong Huh, Kyung-Do Han, Hye Ryoun Jang

**Affiliations:** Division of Nephrology, Department of Medicine, Samsung Medical Center, Sungkyunkwan University School of Medicine, Seoul, Republic of Korea; Department of Statistics and Actuarial Science, Soongsil University, Seoul, Republic of Korea; Division of Nephrology, Department of Medicine, Samsung Medical Center, Sungkyunkwan University School of Medicine, Seoul, Republic of Korea; Division of Nephrology, Department of Medicine, Samsung Medical Center, Sungkyunkwan University School of Medicine, Seoul, Republic of Korea; Division of Nephrology, Department of Medicine, Samsung Medical Center, Sungkyunkwan University School of Medicine, Seoul, Republic of Korea; Division of Nephrology, Department of Medicine, Samsung Medical Center, Sungkyunkwan University School of Medicine, Seoul, Republic of Korea; Department of Statistics and Actuarial Science, Soongsil University, Seoul, Republic of Korea; Division of Nephrology, Department of Medicine, Samsung Medical Center, Sungkyunkwan University School of Medicine, Seoul, Republic of Korea

**Keywords:** fatty liver disease, kidney transplant, metabolic dysfunction-associated steatotic liver disease, metabolic syndrome, steatotic liver disease

## Abstract

**Introduction:**

Metabolic dysfunction–associated steatotic liver disease (MASLD) has been established to classify steatotic liver disease more accurately. We investigated the association between MASLD and long-term kidney transplant outcomes.

**Methods:**

Kidney transplant recipients who underwent national health screening programs from 2009 to 2017 and with follow-up until 2021 were identified using the Korean National Health Insurance Service data. MASLD was defined based on cardiometabolic criteria and the fatty liver index.

**Results:**

Among 8326 kidney transplant recipients, 2027 (24.3%) had MASLD, 25 (0.3%) had MASLD with increased alcohol intake, and 419 (5.0%) had MASLD with other liver diseases. The incidence rates of graft failure (17.5 vs. 11.3, *P* < .001) and mortality (12.5 vs. 8.9 per 1000 person-years, *P* < .001) were significantly higher in the MASLD group than in those without steatotic liver disease. After adjusting for demographics, lifestyle, comorbidities, estimated glomerular filtration rate, proteinuria, and acute rejection, the adjusted hazard ratios for graft failure and mortality were 1.23 (95% confidence interval, 1.03–1.46) and 1.03 (95% confidence interval, 0.85–1.25), respectively.

**Conclusions:**

This study identified MASLD as a significant risk factor for graft failure in kidney transplant recipients, highlighting the need for MASLD screening and management in kidney transplant recipients.

## Graphical Abstract

**Graphical Abstract:**
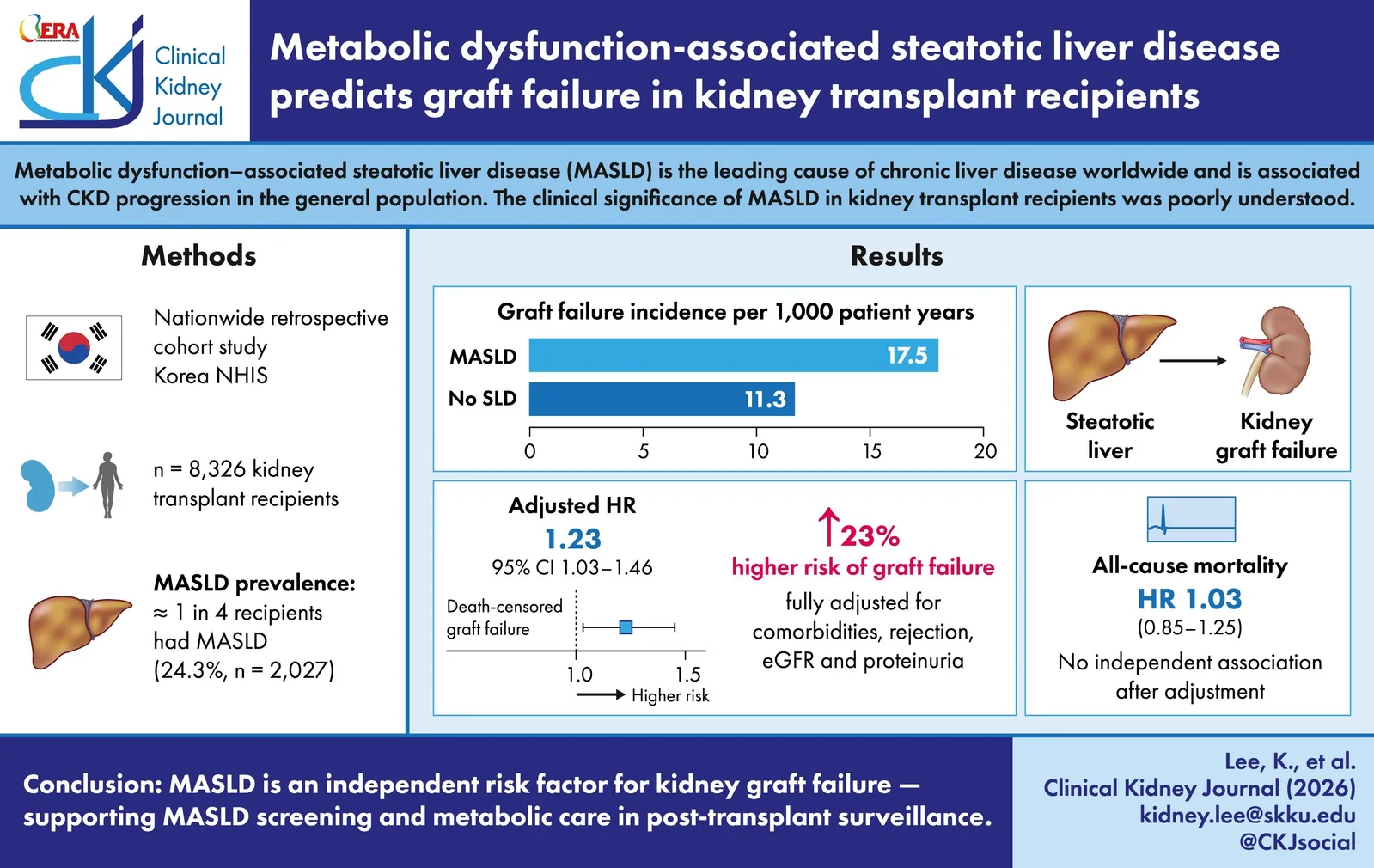

KEY LEARNING POINTS **What was known:**

- MASLD is the most common chronic liver disease worldwide and is strongly associated with cardiovascular disease and chronic kidney disease progression in the general population.
- Kidney transplant recipients are particularly vulnerable to metabolic derangements due to immunosuppressive therapies, yet the prevalence and clinical significance of MASLD in this population remained largely unknown.
- Previous studies on post-transplant steatotic liver disease were limited to small single-center cohorts and did not evaluate hard clinical endpoints such as graft failure using the updated SLD nomenclature.

**This study adds:**

- Approximately one in four kidney transplant recipients (24.3%) had MASLD, demonstrating a substantial disease burden in this population based on a nationwide cohort of over 8000 patients.
- MASLD was independently associated with a 23% increased risk of death-censored graft failure after comprehensive adjustment for demographics, lifestyle, comorbidities, kidney function, proteinuria, and rejection.
- The association between MASLD and graft failure was consistent across subgroups defined by age, sex, and metabolic risk factors, with no significant effect modification observed.

**Potential impact:**

- These findings support incorporating MASLD screening into routine post-transplant surveillance protocols for kidney transplant recipients to identify those at elevated risk of graft failure.
- Targeted management of hepatic metabolic health, through lifestyle modifications or emerging pharmacotherapies, may represent a novel strategy to improve long-term kidney allograft survival.
- The application of the updated SLD nomenclature, including MASLD and MetALD classifications, provides a more precise framework for risk stratification in transplant clinical practice.

## INTRODUCTION

Metabolic dysfunction–associated steatotic liver disease (MASLD) is a recently updated definition that replaces the term “nonalcoholic fatty liver disease” (NAFLD) and more accurately reflects the metabolic drivers underlying this condition and its systemic consequences [1]. Paralleling the global increase in diabetes and metabolic syndrome, MASLD is becoming increasingly prevalent and is now the most common chronic liver disease worldwide [2, 3]. Importantly, MASLD is strongly associated with increased risks of cardiovascular disease and chronic kidney disease (CKD), underscoring its systemic impact [2, 4].

Kidney transplantation (KT) is the optimal treatment for end-stage kidney disease (ESKD), offering substantial improvements in patient survival and quality of life [5]. Despite incremental improvement in kidney allograft survival over recent decades [6], long-term graft survival remains limited [7, 8]. Organ transplantation per se poses significant metabolic challenges, with immunosuppressive therapies and comorbidities frequently causing or worsening metabolic derangements that may compromise allograft survival [9]. Although the impact of traditional risk factors on graft outcomes is well-studied, the potential role of MASLD, a driver of systemic inflammation and metabolic stress, in KT outcomes remains poorly understood. Considering the shared metabolic disturbances and systemic inflammation linking MASLD and CKD [4], it is plausible that MASLD negatively affects allograft survival in KT recipients.

Given this biological plausibility and the limited data on the prevalence and prognostic significance of MASLD in KT recipients, understanding this relationship using the recently revised steatotic liver disease (SLD) criteria is needed to identify individuals at high risk and develop targeted management strategies. Therefore, we aimed to investigate the association between MASLD and long-term KT outcomes, specifically graft failure and all-cause mortality, using a large-scale nationwide health database.

## MATERIALS AND METHODS

### Data source and study design

We conducted a nationwide retrospective cohort study using data from the Korean National Health Insurance Service (NHIS) database, which provides universal healthcare coverage to ∼97% of the South Korean population. This database includes comprehensive information on demographics, socioeconomic status, medical claims, prescription data, and health screening results. The solid-organ transplant cohort was established based on the beneficiary charges claimed by the healthcare providers [10]. Within the solid-organ transplant cohort, adult KT recipients (age ≥20 years) were accurately identified using the NHIS code, R3280, specific for KTs performed between 2004 and 2017. Patients with concurrent NHIS procedure codes for other solid-organ transplantations, such as pancreas, liver, or heart transplantation, during the same admission for KT were excluded.

### Study population

Of the 26 087 adult KT recipients, the present cohort comprised the 9129 who underwent national health examinations between 2009 and 2017. The date of enrollment was 1 January 2009, and the follow-up ended on 31 December 2021. Recipients diagnosed with liver cancer (*n* = 21), those who underwent liver transplantation (*n* = 108), those with SLD without cardiometabolic risk factors (specific-etiology or cryptogenic SLD) or those with excessive alcohol intake (alcohol intake ≥50 g/day for female, ≥60 g/day for male) (*n* = 11), those with missing values (*n* = 411), those who experienced graft failure before health examination (*n* = 136), and those who experienced graft failure or died within 1 year of lag time after the index date (*n* = 116) were excluded.

### Definition of MASLD

MASLD was defined according to recently established criteria requiring evidence of hepatic steatosis in conjunction with cardiometabolic risk factors [1]. Hepatic steatosis was identified using the fatty liver index, which incorporates waist circumference, body mass index (BMI), serum triglycerides, and gamma-glutamyl transferase (GGT) levels [11, 12]. Recipients with MASLD with increased alcohol intake (average 20–50 g/day for female, 30–60 g/day for male) were categorized separately as MASLD and increased alcohol intake (MetALD) according to the revised nomenclature consensus [1]. If recipients with MASLD had other concomitant liver diseases, they were categorized separately as MASLD with other liver diseases. The presence of concomitant liver diseases was defined according to the following International Classification of Diseases (ICD-10) codes: drug-induced liver disease (K71), viral hepatitis (B15–19, B00.8, and B25.1), hepatic veno-occlusive disease (I82), liver abscess (K75.0 and A06.4), hemochromatosis (E83.1), Wilson disease (E83.0), alpha-1 antitrypsin deficiency (E88.0), autoimmune hepatitis (K75.4), primary biliary cholangitis (K74.3 and K74.4), other cholangitis (K83 and K83.0A), and glycogen storage disease (E74). The first national health examination performed after KT during 2009–17 was used as the index date, and all fatty liver index components (waist circumference, BMI, triglycerides, and GGT) and covariates were obtained from that single index examination.

### Study outcomes: graft failure and mortality

The outcomes of interest were death-censored graft failure (defined as a return to dialysis or preemptive re-transplantation) and all-cause mortality. Graft failure was determined using the Health Insurance Review and Assessment Service procedure codes for dialysis or re-transplantation. Graft failure based on dialysis code was defined as continuous dialysis for 3 months post-KT. Mortality was determined using data from the Korean National Death Registry.

### Covariates

Data on demographics (age, sex, and income level), lifestyle factors (smoking status, alcohol consumption, and physical activity), medical history, anthropometric measurements (height, weight, and waist circumference), blood pressure, and laboratory test results were collected. Laboratory data included fasting glucose, total cholesterol, high-density lipoprotein cholesterol, low-density lipoprotein cholesterol, triglyceride, alanine aminotransferase (ALT), aspartate aminotransferase (AST), GGT, serum creatinine, and proteinuria. Serum creatinine levels were standardized by isotope dilution mass spectrometry. The estimated glomerular filtration rate (eGFR) was calculated using the Chronic Kidney Disease Epidemiology Collaboration equation [13]. Proteinuria was assessed using urine dipstick testing, with a dipstick reading of 1+ or greater indicating the presence of proteinuria. BMI was calculated as weight in kilograms divided by height in meters squared (kg/m²).

Diabetes mellitus was defined as either a fasting glucose level ≥126 mg/dl or at least one claim for ICD-10 codes E11–E14 accompanied by a prescription for any antidiabetic medication, including insulin. Hypertension was defined as a systolic blood pressure ≥140 mmHg, diastolic blood pressure ≥90 mmHg, or the presence of a claim code for antihypertensive medication prescription under ICD-10 codes I10–I13 or I15. Low income was defined as being in the lowest quartile of the population based on NHIS premium contribution or being a beneficiary of the Medical Aid Program. Alcohol consumption (g/day) was estimated using standardized self-reported questionnaires that assessed drinking frequency, average amount per occasion, and beverage type. Regular physical activity was defined as engaging in vigorous-intensity activity ≥3 days/week for ≥20 min/day or moderate-intensity activity ≥5 days/week for ≥30 min/day, in accordance with NHIS health screening definitions and previous Korean cohort studies. Acute rejection within 1 year after transplantation was defined using claims for antirejection treatment. Specifically, recipients were considered to have experienced acute rejection if, after discharge from the index transplant admission, they were readmitted within 1 year and received any of the following treatments: rituximab, antithymocyte globulin, intravenous corticosteroid pulse therapy (administered for ≥3 consecutive days), intravenous immunoglobulin, or therapeutic plasmapheresis.

### Statistical analyses

The baseline characteristics of the recipients were compared between the groups with and without MASLD using chi-square tests for categorical variables and analysis of variance for continuous variables. Incidence rates were expressed per 1000 person-years. Kaplan–Meier survival curves were generated, and differences were assessed using the log-rank test.

To estimate the hazard ratios (HRs) for the associations between MASLD and study outcomes, we used Cox proportional hazards models, reporting both unadjusted and multivariable-adjusted HRs. The fully adjusted model included covariates for demographics, socioeconomic status, lifestyle factors, comorbidities, eGFR, proteinuria, and acute rejection. BMI and the other fatty liver index components (waist circumference, triglycerides, and GGT) were not entered into the multivariable models, as they are constituents of the index used to define MASLD. Additionally, a competing risk analysis (using the Fine–Gray model) was performed, with death before graft failure as the competing event. The results are presented as HRs with 95% confidence intervals (CIs).

Subgroup analyses and interaction tests were performed to evaluate the consistency of associations across strata defined by sex, age, and metabolic risk factors. Statistical significance was defined as a two-sided *P* value <.05. All statistical analyses were conducted using SAS version 9.4 (SAS Institute, Cary, NC, USA).

### Study approval

The study protocol adhered to the ethical guidelines of the 1975 Declaration of Helsinki and was reviewed and approved by the Institutional Review Board of Samsung Medical Center (IRB no. 2023-01-006). The requirement for informed consent was waived owing to the use of de-identified and routinely collected administrative data. The reported clinical and research activities were consistent with the Principles of the Declaration of Istanbul, as outlined in the “Declaration of Istanbul on Organ Trafficking and Transplant Tourism.” Strengthening the Reporting of Observational Studies in Epidemiology guidelines were used to ensure transparent and complete reporting of this observational study [14].

## RESULTS

**Figure 1:**
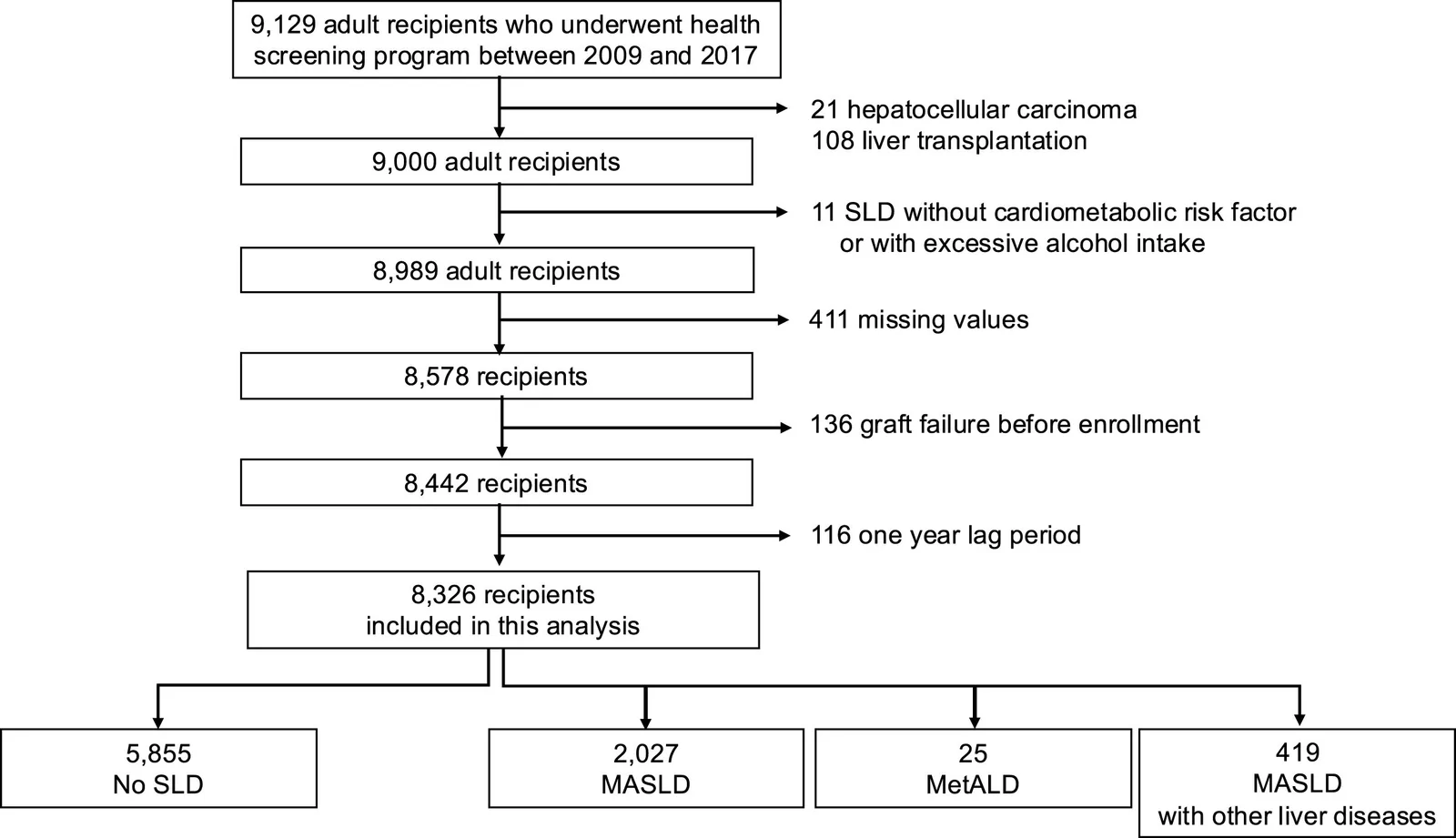
Flowchart of patient inclusion. MASLD, metabolic dysfunction–associated steatotic liver disease; MetALD, metabolic dysfunction–associated steatotic liver disease and increased alcohol intake; SLD, steatotic liver disease.

In total, 8326 KT recipients were included in the study (Fig. 1): 5855 (70.3%) without SLD, 2027 (24.3%) with MASLD, 419 (5.0%) with MASLD with other liver diseases, and 25 (0.3%) with MetALD. The interval between KT and the index health examination, at which SLD status was ascertained, was a median of 2.28 years (IQR, 1.18–4.32; mean, 3.03 ± 2.40 years). The baseline characteristics according to SLD status are presented in Table 1. Recipients with MASLD, MASLD with other liver diseases, and MetALD were older and more likely to be male compared with those without SLD. The prevalence rates of diabetes mellitus, hypertension, and dyslipidemia were significantly higher in the MASLD, MASLD with other liver diseases, and MetALD groups than in the no-SLD group. The proportion of current smokers was higher in the MASLD, MASLD with other liver diseases, and MetALD groups than in the no-SLD group, whereas the proportion of those engaging in regular exercise was comparable across groups. The MASLD, MASLD with other liver diseases, and MetALD groups had higher BMI and waist circumference. Regarding laboratory parameters, AST, ALT, and GGT levels were higher in the MASLD, MASLD with other liver diseases, and MetALD groups. Proteinuria was more prevalent in the MASLD, MASLD with other liver diseases, and MetALD groups than in the no-SLD group, whereas eGFR levels were comparable across all groups. The prevalence of acute rejection within one year after KT was also similar across the SLD groups.

**Table 1:** Baseline characteristics according to SLD status.

	No SLD (*n* = 5855)	MASLD (*n* = 2027)	MASLD with other liver diseases (*n* = 419)	MetALD (*n* = 25)	*P*
Age					<0.001
<40 years	1010 (17.3)	250 (12.3)	43 (10.3)	4 (16.0)	
40–64 years	4543 (77.6)	1641 (81.0)	342 (81.6)	21 (84.0)	
≥65 years	302 (5.2)	136 (6.7)	34 (8.1)	0 (0.0)	
Male	3206 (54.8)	1415 (69.8)	319 (76.1)	24 (96.0)	<0.001
BMI (kg/m^2^)					<0.001
<23	4231 (72.3)	252 (12.4)	72 (17.2)	3 (12.0)	
23–24.9 (overweight)	1165 (19.9)	530 (26.2)	99 (23.6)	7 (28.0)	
≥25 (obesity)	459 (7.8)	1245 (61.4)	248 (59.2)	15 (60.0)	
Waist circumference (cm)	76.0 (7.3)	89.2 (7.5)	89.8 (7.9)	90.7 (7.1)	<0.001
Low income^a^	1481 (25.3)	499 (24.6)	113 (27.0)	9 (36.0)	.454
Current smoker	265 (4.5)	201 (9.9)	45 (10.7)	16 (64.0)	<0.001
Alcohol					<0.001
None	5122 (87.5)	1662 (82.0)	373 (89.0)	0 (0.0)	
Mild (<20 g/day)	697 (11.9)	365 (18.0)	46 (11.0)	0 (0.0)	
Moderate (≥20 to <50 g/day)	32 (0.6)	0 (0.0)	0 (0.0)	25 (100.0)	
Excessive (≥50 g/day)	4 (0.1)	0 (0.0)	0 (0.0)	0 (0.0)	
Regular exercise	1515 (25.9)	477 (23.5)	94 (22.4)	5 (20.0)	.092
Diabetes mellitus	1917 (32.7)	960 (47.4)	228 (54.4)	15 (60.0)	<0.001
Hypertension	3589 (61.3)	1525 (75.2)	340 (81.2)	19 (76.0)	<0.001
Dyslipidemia	3118 (53.3)	1371 (67.6)	283 (67.5)	18 (72.0)	<0.001
Fasting glucose (mg/dl)	104.0 (31.1)	116.4 (40.6)	118.3 (45.2)	139.3 (48.6)	<0.001
Systolic BP (mmHg)	123.9 (14.6)	129.1 (14.8)	128.5 (13.9)	130.6 (13.9)	<0.001
Diastolic BP (mmHg)	77.4 (10.1)	79.9 (10.4)	78.7 (9.5)	82.5 (8.3)	<0.001
eGFR (ml/min/1.73 m^2^)	69.7 (21.1)	67.1 (21.0)	68.1 (20.5)	75.8 (19.4)	<0.001
Proteinuria	460 (7.9)	292 (14.4)	57 (13.6)	7 (28.0)	<0.001
AST (IU/l)	21.7 (6.9)	24.1 (9.1)	25.6 (10.1)	32.9 (23.2)	<0.001
ALT (IU/l)	22.7 (12.0)	46.1 (32.7)	55.0 (46.0)	101.9 (101.5)	<0.001
GGT (IU/l)	18.8 (9.2)	27.2 (15.5)	28.1 (16.1)	28.6 (16.8)	<0.001
Total cholesterol	180.9 (34.9)	190.2 (37.0)	187.8 (39.1)	195.7 (45.0)	<0.001
Triglyceride (mg/dl)	108.7 (46.5)	180.6 (83.5)	179.7 (86.9)	261.9 (159.2)	<0.001
HDL-cholesterol (mg/dl)	61.2 (20.2)	53.2 (20.4)	53.5 (32.7)	55.7 (10.7)	<0.001
LDL-cholesterol (mg/dl)	97.5 (32.4)	100.3 (36.4)	97.8 (46.9)	88.4 (44.8)	.007
Acute rejection	1889 (32.3)	679 (33.5)	143 (34.1)	9 (36.0)	.659

BMI, body mass index; BP, blood pressure; GGT, gamma-glutamyl transferase; MetALD, metabolic dysfunction–associated steatotic liver disease and increased alcohol intake.

Values are presented as *n* (%) or mean (standard deviation).

^a^“Low income” was defined as being in the lowest quartile of the population based on the NHIS premium contribution or as a beneficiary of the Medical Aid Program.

### Assessment of graft failure risk

**Figure 2:**
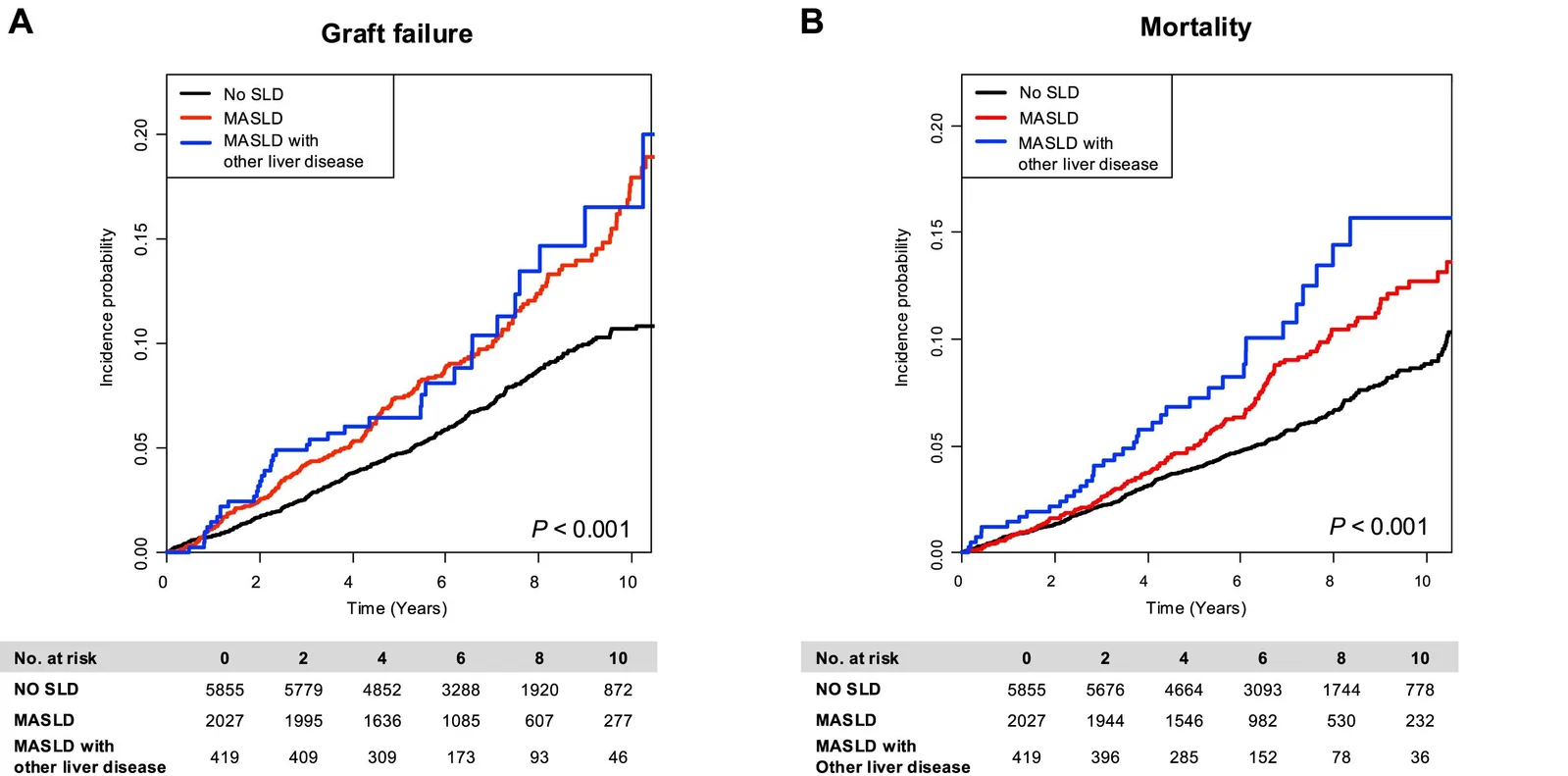
Cumulative incidences of (A) death-censored graft failure and (B) all-cause mortality, stratified by SLD status. Patients were categorized into three groups: no SLD (black line), metabolic dysfunction–associated steatotic liver disease (MASLD; red line), and MASLD with other liver diseases (blue line). Compared with the no-SLD group, the cumulative incidence of both graft failure and mortality was significantly higher in the MASLD and MASLD with other liver diseases groups (log-rank *P* < .001 for both analyses). The MetALD group was not included in the graphical analysis because of insufficient sample size. MASLD, metabolic dysfunction–associated steatotic liver disease; MetALD, metabolic dysfunction–associated steatotic liver disease and increased alcohol intake; SLD, steatotic liver disease.

Graft failure occurred in 214 (10.6%) of 2027 KT recipients with MASLD and in 420 (7.2%) of the 5855 patients without SLD. The overall incidence rates of graft failure were 17.5 and 11.3 per 1000 person-years in the MASLD and no-SLD groups, respectively. Death-censored graft failure rates were significantly higher in the MASLD and MASLD with other liver diseases groups than in the no-SLD group (Fig. 2A, *P* < .001).

In the unadjusted analysis, the MASLD group had a 1.55-fold (95% CI, 1.32–1.83) higher risk of graft failure than the no-SLD group. After adjusting for multiple covariates, the graft failure risk remained consistently higher for the recipients with MASLD: model 1: HR, 1.41 (95% CI, 1.19–1.68; adjusted for age, sex, income, smoking, regular exercise, diabetes, hypertension, dyslipidemia, and duration between KT and health screening); model 2: HR, 1.23 (95% CI, 1.03–1.46; adjusted for age, sex, income, smoking, regular exercise, diabetes, hypertension, dyslipidemia, duration between KT and health screening, eGFR, proteinuria, and acute rejection). When competing risk analysis was performed, with death before graft failure as a competing event, the sub-distribution HRs for graft failure were 1.41 (95% CI, 1.19–1.68) for model 1 and 1.23 (95% CI, 1.03–1.47) for model 2 in the MASLD group (Table 2).

**Table 2:** Hazard ratios for graft failure rate according to SLD status.

	*n* (event)	IR^a^	Unadjusted HR (95% CI)	HR (95% CI)	
				Model 1^c^	Model 2^c^
Graft failure (death-censored)					
No SLD	420	11.3	Ref.	Ref.	Ref.
MASLD	214	17.5	1.55 (1.32–1.83)	1.41 (1.19–1.68)	1.23 (1.03–1.46)
MASLD with other liver diseases	41	18.3	1.67 (1.21–2.30)	1.55 (1.12–2.15)	1.55 (1.12–2.15)
MetALD	1	7.2	0.66 (0.09–4.71)	0.37 (0.05–2.64)	0.40 (0.06–2.86)
Graft failure^b^ (competing risk)					
No SLD				Ref.	Ref.
MASLD				1.41 (1.19–1.68)	1.23 (1.03–1.47)
MASLD with other liver diseases				1.55 (1.12–2.16)	1.55 (1.11–2.16)
MetALD				0.37 (0.05–2.71)	0.40 (0.06–2.82)

CI, confidence interval; HR, hazard ratio; IR, incidence rate; MetALD, metabolic dysfunction–associated steatotic liver disease and increased alcohol intake; Ref., reference group.

^a^Incidence rate is presented as per 1000 person-year.

^b^The sub-distribution hazard of graft failure (competing events for death before graft failure) was used for HR for competing risk analysis.

^c^Model 1 included age, sex, income, smoking, regular exercise, diabetes, hypertension, dyslipidemia, and duration between kidney transplantation and health screening. Model 2 included model 1 covariates, eGFR, proteinuria, and acute rejection.

The MASLD with other liver diseases group was also associated with a higher graft failure rate, with adjusted HRs of 1.55 (95% CI, 1.12–2.15) for model 1 and 1.55 (95% CI, 1.12–2.15) for model 2.

### Assessment of mortality risks

Over the follow-up period, the incidence rates of all-cause mortality were significantly higher in the MASLD group (12.5 per 1000 person-years) than in the no-SLD group (8.9 per 1000 person-years, *P* < .001 by log-rank test) (Fig. 2B).

In the unadjusted analysis, the MASLD group demonstrated a 1.42-fold (95% CI, 1.18–1.71) increased risk of mortality compared with the no-SLD group. However, after adjusting for demographics, lifestyle factors, comorbidities, eGFR, and proteinuria, MASLD was no longer significantly associated with increased mortality risk (model 1: HR, 1.11; 95% CI, 0.91–1.34; model 2: HR, 1.03; 95% CI, 0.85–1.25) (Table 3).

**Table 3:** Hazard ratios for mortality rate according to SLD status.

All-cause death	*n* (event)	IR^a^	Unadjusted HR (95% CI)	HR (95% CI)	
				Model 1^b^	Model 2^b^
No SLD	341	8.9	Ref.	Ref.	Ref.
MASLD	162	12.5	1.42 (1.18–1.71)	1.11 (0.91–1.34)	1.03 (0.85–1.25)
MASLD with other liver diseases	40	16.6	1.95 (1.40–2.70)	1.40 (1.01–1.95)	1.33 (0.95–1.85)
MetALD	5	34.5	4.16 (1.72–10.07)	2.07 (0.84–5.13)	2.00 (0.81–4.96)

CI, confidence interval; HR, hazard ratio; IR, incidence rate; MetALD, metabolic dysfunction–associated steatotic liver disease and increased alcohol intake; Ref, reference group.

^a^Incidence rate is presented as per 1000 person-year.

^b^Model 1 included age, sex, income, smoking, regular exercise, diabetes, hypertension, dyslipidemia, and duration between kidney transplantation and health screening. Model 2 included model 1 covariates, eGFR, proteinuria, and acute rejection.

Those with MASLD with other liver diseases also showed an increased mortality rate compared with the no-SLD group in the unadjusted analysis only (HR 1.95, 95% CI, 1.40–2.70). The adjusted HRs were 1.40 (95% CI, 1.01–1.95) and 1.33 (95% CI, 0.95–1.85) for models 1 and 2, respectively.

### Subgroup analyses

**Figure 3:**
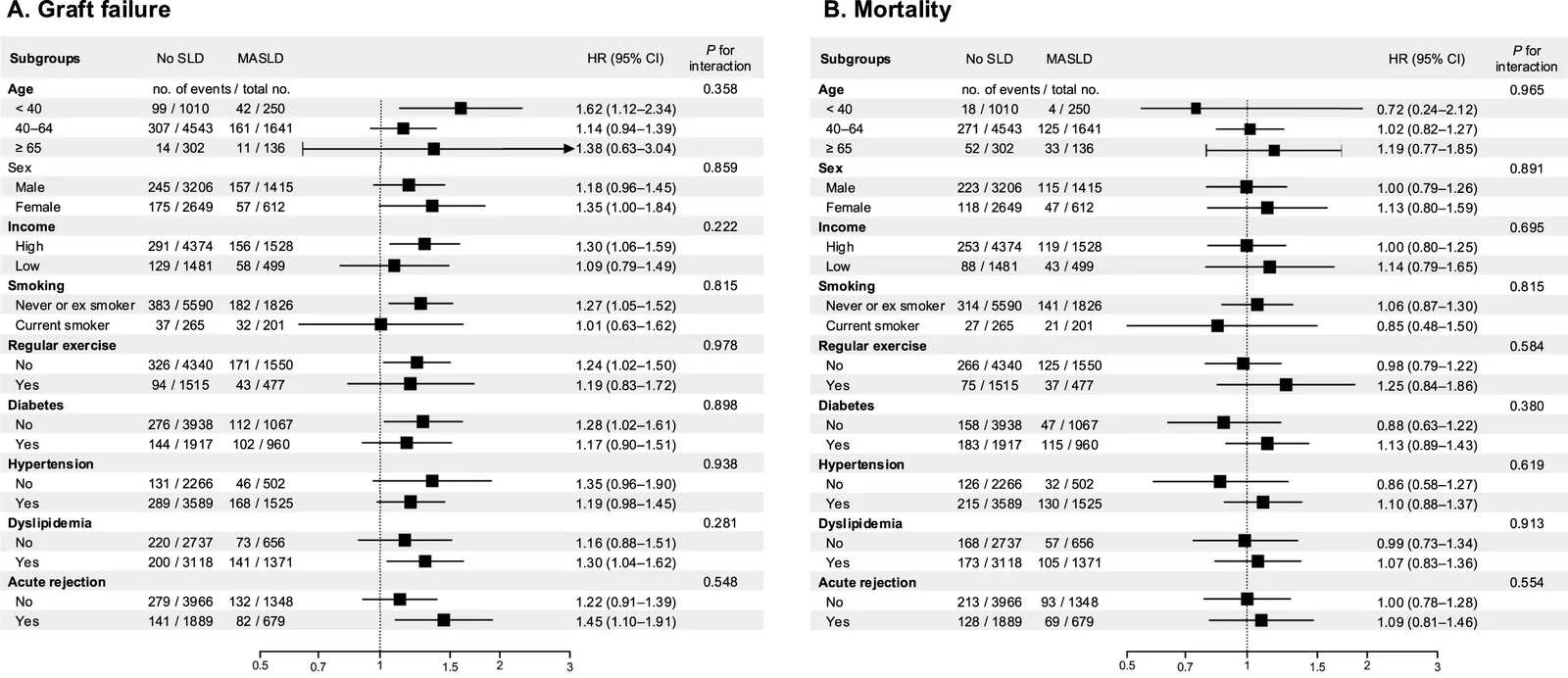
Subgroup analyses for graft failure and all-cause mortality. Demonstrated are forest plots of the risks of (A) graft failure and (B) all-cause mortality. The HRs were adjusted for age, sex, income, smoking, regular exercise, diabetes, hypertension, dyslipidemia, duration between kidney transplantation and health screening, estimated glomerular filtration rate, proteinuria, and acute rejection. CI, confidence interval; HR, hazard ratio; MASLD, metabolic dysfunction–associated steatotic liver disease; SLD, steatotic liver disease.

The subgroup analyses for graft failure and mortality are presented in Fig. 3. There was no significant interaction between MASLD and any subgroup for either outcome (all *P* > .05).

## DISCUSSION

In this nationwide cohort study of more than 8000 KT recipients, we identified a 24% prevalence of MASLD and demonstrated that it independently predicted kidney allograft failure. This association persisted even after comprehensive adjustment for demographic, clinical, and metabolic confounders. Although some single-center studies have reported variable MASLD prevalence in KT recipients [15–17], our study represents the first large-scale investigation to establish both the prevalence and prognostic significance of MASLD in this population.

Despite limited studies on post-transplant SLD in non-liver organ transplant recipients, de novo NAFLD frequently occurs after liver transplantation for non-NAFLD chronic liver diseases [18]. The potential contributors to de novo NAFLD include post-transplant weight gain, sarcopenic obesity, post-transplant diabetes mellitus, and immunosuppressant use, particularly corticosteroids and calcineurin inhibitors [19]. Given that KT recipients experience similar metabolic and pharmacological insults, these mechanisms are likely relevant to KT recipients [19]. Calcineurin inhibitors exacerbate metabolic dysfunction through multiple pathways, including reduction of pancreatic β-cell proliferation and insulin secretion, inhibition of hepatic triglyceride lipase, and impairment of bile acid synthesis [20–22]. Corticosteroids also promote hepatic steatosis through multiple mechanisms [23, 24]. The synergistic effects of post-transplant sarcopenic obesity further amplify hepatic lipid accumulation [19, 25]. Thus, a significant proportion of MASLD cases in our cohort likely represents either aggravation of preexisting SLD or de novo disease after KT. Future longitudinal studies with pretransplant baseline assessments are warranted to precisely determine changes in SLD and de novo disease occurrence.

The mechanistic link between MASLD and renal dysfunction is well-established in non-transplant populations. Multiple studies have identified NAFLD as an independent risk factor for incident CKD, with meta-analyses demonstrating an increased risk of CKD development and accelerated eGFR decline [26–28]. Although no previous studies have directly examined graft failure as an outcome, one study using transient elastography found an inverse correlation between hepatic steatosis severity and eGFR in KT recipients [15], which is consistent with the findings of the present study. Taken together, our results extend previous research that has primarily linked MASLD to the development and progression of native kidney CKD [4], suggesting that the detrimental effects are not confined to the native kidney but may compromise kidney allograft function over time.

Several plausible mechanisms may explain the association between MASLD and graft failure. MASLD is not a localized liver disease but a systemic condition, and it shares several pathogenic mechanisms with graft dysfunction, such as chronic low-grade inflammation, activation of the renin-angiotensin-aldosterone system, endothelial dysfunction, a chronic procoagulant state, and profound insulin resistance [2], suggesting a possible bidirectional relationship between MASLD and graft dysfunction. Proinflammatory cytokines and adipokines released from steatotic liver tissue and dysfunctional adipose tissue may potentiate the alloimmune response against the kidney graft. The baseline post-transplant inflammatory state could be amplified by the metabolic inflammation of MASLD, promoting subclinical inflammation, interstitial fibrosis, and tubular atrophy, leading to chronic allograft dysfunction [4]. Declining graft function, proteinuria, and post-transplant metabolic derangements may further aggravate insulin resistance and hepatic steatosis. Given the emerging understanding of organ crosstalk in kidney injury [29], the precise mechanism by which MASLD compromises kidney graft function is a topic of future study.

The higher prevalence of proteinuria in the MASLD groups most plausibly reflects metabolic and hemodynamic kidney injury, given the greater burden of diabetes, hypertension, and obesity in these recipients. Comparable eGFR values and prevalence of acute rejection across groups further support a metabolic rather than an immune-mediated origin. Although native kidneys are end-stage and usually contribute little urine, those with diabetic nephropathy can remain non-atrophic and proteinuric, and such cases are expected to be more frequent in the MASLD groups [30, 31].

Although the MASLD group showed a markedly higher mortality in the unadjusted analysis, no independent association was observed between MASLD and all-cause mortality after adjustment for confounding variables. Although numerous epidemiological studies have demonstrated increased mortality in patients with NAFLD, whether NAFLD independently increases mortality risk remains controversial [32, 33]. Previous research suggests that excess mortality may be primarily driven by components of metabolic syndrome rather than hepatic steatosis per se [32]. Given the increased cardiovascular mortality burden and extensive comorbidities characteristic of KT recipients [34], the majority of deaths in our cohort were likely attributable to underlying metabolic disturbances and preexisting conditions rather than to liver steatosis itself.

One of the most significant changes in the revised SLD definition was the establishment of MetALD as a new diagnostic category [1], recognizing that a substantial proportion of individuals with MASLD consume moderate amounts of alcohol, representing overlapping biological processes that contribute to both NAFLD and alcohol-associated liver disease, which were not captured by previous definitions [35]. However, our study identified only a limited number of patients meeting the MetALD criteria. This small sample size precluded a meaningful analysis of the associations between MetALD and either graft failure or mortality outcomes. This finding likely reflects the unique lifestyle characteristics of KT recipients who undergo health monitoring and generally maintain minimal alcohol consumption. Alternatively, social desirability bias may have led to underreporting [36], because alcohol consumption data relied exclusively on self-reported questionnaires.

This study has some limitations. First, the retrospective study design precludes definitive causal inference, and residual confounding from unmeasured variables such as specific immunosuppressive regimens, doses, and genetic predispositions cannot be excluded. Second, the diagnosis of MASLD was based on the fatty liver index, a surrogate marker, rather than on definitive methods, such as imaging or liver biopsy, potentially leading to misclassification. However, considering that abdominal ultrasound in KT recipients lacks reliability, the fatty liver index was suggested as a reliable predictor of NAFLD in this population [37]. Another limitation was the lack of data on the severity of liver fibrosis, which is a key predictor of outcomes, thus limiting our ability to stratify risk based on the spectrum of liver disease. Future prospective studies should use precise staging modalities, such as vibration-controlled transient elastography or magnetic resonance elastography [38, 39], to validate our findings and assess the impact of fibrosis severity on graft outcomes. Additionally, the inability to distinguish between transplantations from deceased and living donors, owing to database constraints, represents another limitation. Deceased-donor transplant recipients typically experience longer dialysis duration and greater exposure to the metabolic consequences of ESKD than living-donor recipients [40]. The interaction among MASLD, transplant outcomes, and prevalence according to donor type warrants investigation in future studies. In addition, because the index assessment was the first national health examination performed after transplantation, pretransplant screening data were unavailable. We therefore could not characterize pretransplant metabolic disorders such as masked diabetes mellitus or metabolic syndrome, which may be common during the uremic period and may have contributed to the post-transplant MASLD phenotype. Lastly, our cohort comprised exclusively Korean (Asian) KT recipients. Therefore, the findings might not be generalizable to other racial or ethnic populations, in whom the prevalence of MASLD, body-composition phenotypes, and the diagnostic performance of the fatty liver index may differ.

Despite these limitations, our study has several strengths, including its large nationwide cohort design, which captures real-world outcomes across diverse clinical settings, a long-term follow-up period, and comprehensive adjustment with competing risk analyses. More importantly, we describe the robust association between MASLD and hard clinical endpoints, specifically graft failure, rather than surrogate markers such as eGFR decline or creatinine doubling. By applying the new MASLD nomenclature and including the MetALD classification, we provide an updated assessment of these definitions in the KT population.

In conclusion, our study identifies MASLD as a significant independent risk factor for graft failure in KT recipients. This finding underscores the important role of the liver–kidney axis in the post-transplant setting, suggesting that hepatic metabolic health is linked to renal allograft function and survival. However, the modest effect size warrants cautious interpretation when applied to individual patients. With the updated definition of SLD, our results suggest that MASLD is a significant factor in the risk assessment for post-transplant care of KT recipients. Future interventional studies are warranted to determine whether targeted MASLD management, through intensive lifestyle modifications or emerging pharmacotherapies, can enhance long-term kidney graft survival and improve overall transplant outcomes.

## Data Availability

The data underlying this article will be shared on reasonable request to the corresponding author.
